# Sharing Histories—a transformative learning/teaching method to empower community health workers to support health behavior change of mothers

**DOI:** 10.1186/s12960-017-0231-2

**Published:** 2017-08-23

**Authors:** Laura C. Altobelli

**Affiliations:** 1 Future Generations University, Franklin, USA; 2 Future Generations, Lima, Peru; 3 Universidad Peruana Cayetano Heredia, Lima, Peru

**Keywords:** Sharing histories, Autobiographical memories, Narrative communication, Health behavior change, Health promotion, Community health workers, Community health worker training, Health promoter training, Community health programs, Global health, International health, Maternal and child health, Maternal health, Child health, Community participation, Women’s empowerment, Developing countries

## Abstract

**Background:**

One of the keys to improving health globally is promoting mothers’ adoption of healthy home practices for improved nutrition and illness prevention in the first 1000 days of life from conception. Customarily, mothers are taught health messages which, even if simplified, are hard to remember. The challenge is how to promote learning and behavior change of mothers more effectively in low-resource settings where access to health information is poor, educational levels are low, and traditional beliefs are strong.

**Methods:**

In addressing that challenge, a new learning/teaching method called “Sharing Histories” is in development to improve the performance of female community health workers (CHWs) in promoting mothers’ behaviors for maternal, neonatal and child health (MNCH).

**Results:**

This method builds self-confidence and empowerment of CHWs in learning sessions that are built on guided sharing of their own memories of childbearing and child care. CHWs can later share histories with the mother, building her trust and empowerment to change. For professional primary health care staff who are not educators, Sharing Histories is simple to learn and use so that the method can be easily incorporated into government health systems and ongoing CHW programs.

**Conclusions:**

I present here the Sharing Histories method, describe how it differs from other social and behavior change methods, and discuss selected literature from psychology, communications, and neuroscience that helps to explain how and why this method works as a transformative tool to engage, teach, transform, and empower CHWs to be more effective change agents with other mothers in their communities, thereby contributing to the attainment of the Sustainable Development Goals.

## Introduction

One of the keys to improving health globally to meet the Sustainable Development Goals (SDGs) [1] is adoption by mothers of healthy home practices for better nutrition and illness prevention. Interventions to improve health literacy are recognized as essential for reaching the SDGs [2]. The challenge is acute in settings where access to health information is poor, educational levels are low, and traditional beliefs are strong. Customarily, mothers are taught health lessons that even if simplified are hard to remember. Especially in traditional low-resource settings, a transformative method for applying cultural competence in health promotion efforts is needed to achieve sustainable change of health knowledge and behavior that will improve maternal, neonatal and child health (MNCH) [3, 4].

## Background

The training and deployment of community health workers (CHWs) is globally recognized as a key strategy to close the gap between formal health services and communities [5, 6]. CHWs are part of the World Health Organization (WHO) Global Strategy for Human Resources [7, 8], and WHO is calling for research on implementation of CHW programs given their growing recognition as an integral component to reach impact and equity goals [9].

A systematic review of 82 studies on CHW performance showed CHWs to have promising impact on immunization coverage, breastfeeding, TB treatment, and reducing child morbidity and mortality, when compared to standard care [10]. The 2012 CHW Evidence Summit concluded that the existing empirical data base was not sufficient to identify factors that are likely to improve CHWs’ performance sustainably [11]. More recent systematic reviews identify CHW program design factors that could improve results such as birth outcomes [12, 13]. Others have mixed findings on what influences CHW success [14, 15]. Much work is still required to scale up CHW programs.

Evidence is needed to identify the best training methodologies and materials to improve the efficacy of knowledge-sharing of CHWs [3, 16]. Lack of evidence may be impeding more widespread deployment of CHWs in countries that would benefit from them to reduce maternal, neonatal, and child morbidity and mortality [12]. Also needed are CHW role definitions, supervision, incentives, and system factors that provide a sustainable structure for CHW training and support [17, 18].

Much of the literature on health communications in low-resource settings looks at specific behaviors or treatments taught to mothers, while much less has looked at how to address intrinsic cultural beliefs so that behavior change and health impacts are sustainable. A review of evidence on caregiver behavior change for MNCH identifies available behavior change tools such as “multiple caregiver contacts during antenatal and early childhood periods, motivating caregiver behavior, achievable parenting goals,” while noting that these have not been widely used in global health nor are there comparative effectiveness trials needed for their scale-up [19]. Most of these “tools” assume the use of heuristic information to teach mothers; heuristics being proven facts simplified to their essence with intent to ensure that information is more easily remembered [20]. I surmise that much health education for low-literacy populations suffers from a heuristic orientation that does not adequately address cultural underpinnings of learners’ beliefs and behaviors and is thus at risk of being ineffective.

Health care interventions that can be delivered by CHWs to improve health outcomes have been well identified [14, 21]. However, most reports neither discuss how CHWs are taught to carry out recommended interventions nor discuss how CHWs can communicate effectively with mothers by addressing the beliefs, attitudes, and self-esteem that determine maternal capabilities [22]. An extensive literature review of social and behavior change (SBC) communication trials to reduce child stunting concluded that simply increasing knowledge and awareness of correct nutrition practices does not lead to sustained behavior change [23].

The two leading strategies for MNCH improvement with CHWs are Participatory Women’s Groups (PWGs) [24] and Care Groups [25]. PWGs use a set of methods to transmit messages to mothers in group meetings using a “community action cycle” [26]. The methodology is promoted by WHO and UNICEF [27, 28]. Care Groups use a structured approach to training CHWs in small groups, who then teach mothers [29]. In both PWG and Care Group strategies, the face-to-face dynamics with mothers are not clear and thus may be hard to replicate.

Both PWGs and Care Groups have been shown to achieve positive results on MNCH indicators [30–35], but one observer concluded that both strategies, so far, do not naturally fit into typical government health services in resource-poor settings [36]. This issue is important because governments are responsible for the health care of their populations and are the sole entity that can ensure these programs are scaled up and sustained.

For scaling up CHW programs in government systems, a key aspect is how well primary health care providers can serve as CHW trainers. They tend to rely on medical terminology and heuristic information to train CHWs. Methods for teaching CHWs should accommodate to their educational level, which in most cases is similar to that of mothers with whom they will work.

Most government health systems have yet to solve issues of the training-of-trainers model, CHW training materials, incentives, and supportive supervision. Also, there may be a yet-to-be defined “secret sauce” which is needed to empower women with knowledge, motivation, and increased self-efficacy [36] that could be the next breakthrough to sustainably improve MNCH behaviors [12, 28].

I present here a learning/teaching method for CHWs, called “Sharing Histories,” which is oriented to improving promotion of MNCH in developing communities. I describe briefly how the method differs from other SBC methods and discuss conceptual and empirical literature from psychology, communications, and neuroscience that helps to explain how and why Sharing Histories may work as a transformative learning method.

## Methods

Future Generations^1^ is developing a learning/teaching strategy called Sharing Histories, which is an integrated approach to both CHW training and mothers’ behavior change based on local cultural knowledge and experience. Female CHWs share personal memories of their own experiences during the first 1000 days of life from conception for each of their children. Government health personnel serve as facilitators to help CHWs recall memories, then analyze and build on those memories so CHWs are empowered to change their own behaviors and to use the same method to teach mothers in the home.

### Development of the Sharing Histories method for CHW learning and teaching

The idea was empirically discovered in Afghanistan in 2003 [37]. In order to assess child mortality rates in remote villages, local women were gathered and asked to share detailed accounts of each of their pregnancies, with probing to identify any deaths. Beliefs, practices, problems, and successful health actions, both traditional and modern, were brought out for discussion through the shared histories. Participants were deeply fascinated to hear each other’s stories and became highly enthused and empowered to ask questions and learn more about each situation.

This observation led to development of a strategy and field tests in collaboration with the Afghan Ministry of Health (MOH). Older women trained as CHWs became empowered to replicate learning with other women, convincing them to take charge of their family’s health and to use available health services. Two years after the initial pilot ended, the CHWs continued the work demonstrating a sustainability that had not been seen before. A retrospective evaluation showed a 46% reduction in child mortality [37]. Based on the evidence, the Afghan MOH scaled up the CHW training strategy to 13 provinces, achieving an increase in health coverage by 77% [38, 39].

Adaptation and application of the method was soon conducted in 40 villages in Himalayan valleys of Arunachal Pradesh in East India [40].

### Adaptation and pilot testing of the Sharing Histories method in rural Peru

Future Generations is adapting and testing this community learning methodology elsewhere, first with a small cluster-randomized controlled trial in 28 rural indigenous villages of extreme poverty in the high Andes Mountains of Peru [41].

A second larger field test was conducted also in Peru with a cluster-randomized controlled trial in two study groups distinguished by type of CHW teaching method used. Outcomes measured were maternal knowledge and practice and child growth (manuscript in preparation).

### How the Sharing Histories method works

The Sharing Histories method has five phases through which female CHWs learn key behaviors and competencies to share knowledge with other mothers effectively. Learning for each modular topic follows steps described in user-friendly facilitators’ manuals, each corresponding to a specific flipchart (Table 1).

**Table 1:** Phases of community health worker training and deployment using the Sharing Histories teaching/learning method

Phase I	CHW share memories of experiences in the first 1 000 days of each of their children
Phase II	Cultural beliefs and practices are listed and discussed with a facilitator
Phase III	Key behaviors on picture cards (flipcharts) are learned in the context of shared memories
Phase IV	CHW practice teaching mothers with picture cards (flipcharts) and sharing histories
Phase V	CHW visit pregnant mothers, newborns, and children to teach, monitor, and refer

In the first phase, each CHW shares her memories on a specific topic for each of her children in guided sessions with a trainer (primary health care provider) in monthly workshops. During history sharing, no feedback is given to the CHWs. A list of probing questions is provided to facilitate history-telling if needed. A helper notes down key points on each of the CHWs’ experiences (Table 2).

**Table 2:** Seven community health worker training topics on key knowledge and practice for the first 1000 days of life

Pregnancy	
Birth and postpartum	
Newborns	
Breastfeeding	
Complementary feeding and growth	
Infant diarrhea and hygiene	
Infant pneumonia	

In the second phase, cultural beliefs and practices mentioned in the histories are listed. Led by the trainer, each point on the list is discussed in depth as to whether it is helpful, neutral, or harmful to the mother, newborn, or child, and why. This exercise leads to the emergence of a new collective understanding among the CHWs.

The third phase is a discussion of CHW memories in relation to key knowledge and practices within each training topic, viewing picture cards on the corresponding flipchart. This process serves the double purpose of helping CHWs learn and internalize correct information and teaching them how to use the method (Sharing Histories) and tools (flipcharts) to teach mothers in the home.

In the fourth phase, CHWs practice a skill (such as handwashing) or practice teaching mothers in skits using the flipcharts and Sharing Histories under the supervision of trainers. After each session, learning is evaluated with game-like exercises.

For the fifth phase, CHWs conduct monthly home visits to pregnant mothers and those with a child up to 2 years of age, sharing histories to establish trust and using flipcharts to teach mothers key knowledge and practices in the context of their memories. CHWs also monitor maternal behaviors and detect danger signs for referral using checklists.

## Theoretical and empirical evidence that supports the effects of Sharing Histories

Program approaches for global health promotion are generally not explained (or designed) in terms of the key processes through which child caregivers decide, consciously or not, to change their behavior. *Heuristic* learning, based on learning optimal behaviors, underlies most traditional health education and communication [20, 42]. This is one of two fundamentally different ways of knowing, the other being *narrative* [43].

### Use of “narrative communication” for health behavior change

Narrative communication has emerged in the past decade as an important tool for health behavior change because it is a “basic mode of human interaction” [42]. Health communicators are increasingly using techniques such as entertainment education, storytelling, testimonials, and a range of similar means to reach these goals, which are all in the developing definition of narrative communication [44].

In practical terms, narrative communication is the telling of a story [42]. Five specific types of stories have been identified, each used for different communication purposes to promote health behaviors: *official stories*, constructed to tell an innocuous version of events, *invented stories*, *firsthand experiential stories*, *secondhand stories* of others that are retold, and *culturally common stories* that represent a specific cultural context [45]. *Firsthand experiential stories* as well as *secondhand stories* are relevant to Sharing Histories.

Most research on narrative communication for behavior change is found in the fields of psychology and communications, with several theories explaining its behavior-change effects [42]. “Dual-processing models” of persuasion such as the *elaboration likelihood model* (ELM) [46] and *heuristic systematic model* [47] have been used to explain how people are persuaded by narratives, whether fictional or non-fictional. Personal involvement in the topic of the narrative, such as personal narratives shared by CHWs on their childbirth and child care experiences, generally results in more appropriate acceptance of messages [42].

Other behavior change theories on narrative communication that support the effect of Sharing Histories include the Social Cognitive Theory [48], the Precaution Adoption Process [49], and the Theory of Reasoned Action [50]. A commonality of these theories is that the narrative process works best when the person relating the narrative is viewed as culturally similar to the listeners who will then change their behavior.

Hinyard and Kreuter [42] compared studies on the effects of narrative and non-narrative communication on persuasion to change health behaviors, where non-narrative referred to statistical evidence (heuristic learning). Two of three meta-analyses of studies making this comparison found that narrative communication was by far the more persuasive [51, 52]. The third found statistical evidence as more persuasive [53], but its exclusion criteria left out a number of studies included in the former reviews [42]. Most of the studies were on the US populations. In its ability to support generalization to varied global populations, narrative communication seems to be most effective in population sub-groups having a strong oral tradition [42], such as some African-American populations and American-Indian populations that apply narrative storytelling as a strategy for cultural survival [54, 55].

Narrative communication is thus a promising tool for behavior-change communication, with more research needed on narrative versus non-narrative communication under different conditions and cultural settings [42].

### Use of “autobiographical memories” for health behavior change

Autobiographical memory has been an area of research in the psychological literature for over two decades, showing it to be intricately related to the narrative sharing of *firsthand experiential* and *secondhand stories.* The literature on this topic provides a plausible underlying explanation of how Sharing Histories helps women learn how effectively to convince other women to change behaviors.

Much memory research has focused on how, how much, and how accurately people remember their past, but there is growing theory and evidence that memory can be used for behavior change. The utility of memory has been shown to fit into three categories: *self-function* (continuity of the self), *social function* (developing and maintaining social bonds), and *directive function* (guiding present and future behaviors) [56–58].

Research on the directive function of autobiographical memories on future behavior is compelling [57, 59–62]. The directive function can be related to health behavior change if, in fact, one of the major functions of autobiographical memories is to provide flexibility in the formulation and updating of one’s own “rules” that allow individuals to understand the past and to guide future behavior [63, 64]. The more specific a memory, the more likely it is to direct future behavior [59]. Functions of memory may differ if they are positive or negative memories. Positive memories may serve more for the self-function and social functions, while negative memories are more likely to serve directives in life [57]. For example, the sharing of negative memories of pregnancy complications or child illness would serve to help change future behavior.

The directive effect of memories has been tested by comparing memories with high closure versus those with low closure. A psychologically low-closure memory “feels more subjectively as if it is part of the present rather than the past” in that the memory represented a failure to achieve a goal [65] as in a failure to have a healthy pregnancy or a healthy child. Low-closure memories generate more intense emotion and are recalled with more emotional detail [66]. Activating memory, especially if it has high emotional intensity, predicts future plans and decisions [61, 62]. Empirical research showed that when low-closure memories were recalled, subjects were more likely to act on relevant behavioral opportunities that might “restore a positive sense of self in that domain” [60]. Thus, the recall and discussion by CHWs of emotional memories, especially if they are negative and thus low-closure from their childbearing and child-rearing experiences, are likely to result in positive learning and behavior change in the future.

Pillemer and colleagues showed that memories recounted by other people can serve the same function as one’s own memories [67] although at lower levels of intensity. The sharing of personal histories in a group of CHWs in training would expand the range of memories of key events in childbearing and child-rearing that serve to direct future behavior.

The most basic social function of autobiographical memories is to “provide material for conversation, thus facilitating social interaction” [68]. Nelson states that “the initial functional significance of autobiographical memory is that of sharing memory with other people, and that memories become valued in their own right – not because they predict the future and guide present action, but because they are shareable with others and thus serve a social solidarity function, which is a universal human function with culturally-specific rules” [69]. Sharing memories “can engage the listener and elicit empathic responses, particularly if the listener responds with a personal memory of a similar experience” [56]. Empathy is an important factor in learning and remembering. Trust is a key issue for CHW work in communities [10]. Empathic bonds between women from sharing personal stories could be essential for creating the trust that is fundamental to persuade another to change.

According to Pillemer, the recall of deeply held memories of “momentous events,” such as those surrounding the gestation, birth, and early years of a daughter’s or son’s life, “has psychological functions that go to the core of security and self-confidence in one’s self” [70].

I postulate that both the directive and social functions of autobiographical memories provide a theoretical and empirical underpinning for Sharing Histories as a way to enhance learning and self-confidence for empowerment of women to change health behavior.

Most research on autobiographical memory is on Western populations but has relevance to other cultures. Alea and Wang [71] cite studies from Japan [72], China [73], the Caribbean [74], and Australia [75] that report the relevance of the three-function framework of autobiographical memories.

Recent research in the field of neuroscience provides some of the most compelling evidence of the effects of memory on learning and behavior, addressing the theoretical question of how existing memories interact with new learning. Measures of brain encoding suggest that new learning that overlaps with memories of prior experience can interact to construct integrated knowledge [76]. The authors conclude that understanding of these mechanisms may inform learning in real-world settings such as education.

## Discussion

To the best of my knowledge, the use of autobiographical memory has not been previously reported in the context of a systematic method for CHW teaching or learning in a global setting. I propose that psychological, communications, and neuroscience research on memory and narrative communication provide some underlying concepts that can help us construct more effective behavior change approaches for mothers in resource-poor settings to achieve better child and maternal health. Mothers, especially those with low educational attainment, have special needs to achieve effective learning since *they are the primary caregivers and the first-level health care providers for their children*. Health knowledge and practices can best be transmitted by those who are trusted and are culturally similar. Respected older women, who can be trained and referred to as CHWs, live in the same communities and can best fulfill this role since they can more easily gain mothers’ trust.

To contrast with other methods, a popular SBC field manual provides a series of “tried and true methods” (though without citation of evidence-based literature) that CHWs and others can utilize for community promotion of behavior change [21]. One method most relevant to Sharing Histories is the *guided testimonial* based on the identification of a community member who is correctly practicing a behavior. That person provides personal testimony on the behavior with the assumption that others will adopt it after seeing how satisfied their peer is with that behavior. The difference with our new method is that *guided testimonials* and other comparable techniques attempt to engage the learner with ideal behaviors. They also require significant preparatory work on the part of trainers to identify people who practice the behavior correctly and on the part of the latter to practice presenting the testimonial.

CHW training may fall to professional primary health care personnel who generally neither are trained as educators nor are necessarily fully aware of the cultural underpinnings of local populations. Addressing culture in social and behavior change programs can be seen as a costly and long-term effort requiring in-depth studies and analysis by outside experts. Sharing Histories has the cultural analysis built-in to each learning session and serves as an on-the-spot method for identifying and incorporating cultural issues into CHW learning based on women’s memories. In this way, Sharing Histories is a simple, effective, and low-cost method for primary health care staff to learn cultural patterns from CHWs so they can better teach them.

Sharing Histories does not rely on only positive behaviors to share nor does it need preparation; the material for learning is the raw material of mothers’ memories. All mothers (CHWs) can share their experiences, not just one or two mothers, so the pool of memories is larger for discussion and learning.

Sharing Histories was originally built on the concept of *empowerment*, a term that defies easy definition, but is the focus of Future Generations in their applied “theory of community change” based on community members as the key builders of their own future [77, 78]. Empowerment can be defined as “the capacity of individuals, groups and/or communities to take control of their circumstances, exercise power and achieve their own goals, and the process by which, individually and collectively, they are able to help themselves and others to maximize the quality of their lives” [79]. Program interventions that empower people and communities from the bottom-up rather than with top-down approaches that disempower tend to promote agency and achieve behavior change and more sustainable development [80]. Empowerment of women “through improved health education and information sharing among women and their community members” has been identified as one of the key strategies used by fast-progressing countries to scaling-up interventions for reducing neonatal mortality [81] and is an important component of the post-2015 Sustainable Development Agenda. Empowerment of government staff to implement organizational change and collaboration among multisectoral government services and governance structures may also be essential to promote sustainability.

The Sharing Histories approach to teaching CHWs is culturally relevant because it is grounded in the personal experience and memories of each CHW in her own pregnancy, childbirth, breastfeeding, weaning, and child care experience for each of her children. We are testing this method with female CHWs since only women have the intimate experience and therefore critical memories of the momentous events of pregnancy, childbirth, and infant and child care and feeding. Furthermore, the social trust relationship between the CHW and mothers in the home can best be developed between women.

## Conclusion

The two-part premise here is that (1) a learning/teaching method for use with and by community health workers (CHWs) based on their memories, called Sharing Histories, can be a key intervention that can transform them into more effective change agents and (2) as such change agents, CHWs can better empower mothers for behavior change. Use of narrative communication with autobiographical memories as key elements to train female CHWs holds promise for increasing the efficacy of CHWs to help change mothers’ behaviors and achieve greater health impacts in low-resource settings. Ideally, better learning/teaching methods should be used along with culturally appropriate materials to guide the teaching and monitoring of mothers and children in the home, with community-based supervision of CHWs and support from local health services and local government. Used alone, Sharing Histories could potentially be piggybacked onto CHW programs anywhere.

More implementation research is needed on CHW learning methods that enhance their performance with evidence of impact at scale. Methods should be adapted and tested in different cultural settings, with results disseminated to expand prioritization of CHW training and deployment. Policies and guidelines are needed to support CHWs at scale. The aim is to contribute to achieving the SDGs for vulnerable populations of women and children.

## Abbreviations

CHW: Community health worker
MNCH: Maternal, neonatal, and child health
MOH: Ministry of Health
PWG: Participatory Women’s Group
SBC: Social and behavior change
SDG: Sustainable Development Goal
UNICEF: United Nations Children’s Fund
WHO: World Health Organization

## Footnotes

1 A non-profit educational organization dedicated to sustainable community change: www.future.edu
